# Patients’ perspectives of outcomes after total knee and total hip arthroplasty: a nominal group study

**DOI:** 10.1186/s41927-019-0101-8

**Published:** 2020-01-13

**Authors:** Susan M. Goodman, Bella Mehta, Serene Z. Mirza, Mark P. Figgie, Michael Alexiades, Jose Rodriguez, Peter Sculco, Michael Parks, Jasvinder A. Singh

**Affiliations:** 1 Department of Medicine, Hospital for Special Surgery, 535 E 70th Street, New York, NY 10021 USA; 2 New York, USA; 3 Department of Orthopedic Surgery, Hospital for Special Surgery, 535 E 70th Street, New York, NY 10021 USA; 4 Department of Medicine, University of Alabama, 1802 6th Avenue, Birmingham, AL 35233 USA

**Keywords:** Arthroplasty, Outcome measures, Qualitative research

## Abstract

**Background:**

While total joint replacements (TJR) are frequently performed, there is little qualitative research to define the outcomes most important to patients.

**Methods:**

Patients who had received total hip (THR) or total knee replacements (TKR) participated in 8 nominal groups to answer the question “What result/results matter the most to a patient undergoing/having a knee or hip replacement?” Total 270 votes were allocated.

**Results:**

Eight nominal groups were performed with 45 patients, 6 groups with mean age (71.1 ± 9.3), and 2 with 9 younger patients (mean age 36.8 ± 7.4). All had TJR between 2016 and 2018; overall, 40% were male, 15.6% were Black, and 75% were performed for osteoarthritis. While all groups ranked the same top 3 outcomes, responses varied with age: 1) relief of pain (46% vs. 35% in the young groups); 2) improved function including mobility (29% vs. 18% in the young groups); 3) restored quality of life (13% vs 33% of votes in the younger group).

**Conclusion:**

Relief of pain and restoration of function, and improved quality of life are the 3 outcomes ranked highest by patients, confirming their inclusion in TJR clinical trials.

## Background

Total joint replacement(TJR), including total hip(THR) and total knee replacement(TKR) are effective surgical procedures for patients with advanced symptomatic arthritis [1]. These two procedures are among the most frequently performed surgeries, and utilization is projected to increase over the next decade [2]. However, despite overall clinical improvement, up to 30% of patients report that they are dissatisfied due to insufficient pain relief, inadequate functional improvement, or failure to meet their pre-operative expectations [3, 4]. Clinical trials do not typically include harmonized outcomes measures that would permit data pooling or comparison between groups, making it difficult to analyze unsatisfactory outcomes across groups, so the reasons for the unsatisfactory outcomes are difficult to determine [5, 6]. Non-harmonized outcome measures make meta-analyses more challenging and thus hinder the ability to understand and improve TJR outcomes.

The Outcomes Measures in Rheumatology(OMERACT) Total joint Replacement(TJR) Special Interest Group(SIG) is an international group of stakeholders including rheumatologists, orthopedists, methodologists, physical therapists, and patients. This group has previously developed a core domain set for use in TJR clinical trials performed for end-stage hip and knee arthritis [7]. The six core domains were derived from systematic literature review, consensus stakeholder panels, and surveys, and include pain, function, patient satisfaction, revision, adverse events, and death [8]. While the OMERACT process includes patient research partners, and patients have confirmed the selected domains via survey, the domain selection has not benefitted from open-ended patient input. Although patients have endorsed the domains selected and proposed by the expert-led OMERACT TJR Special Interest Group, to our knowledge, there is no qualitative work eliciting the domains of importance from patients.

Nominal Group Technique(NGT) is a highly structured and efficient method for achieving consensus in small face-to-face discussion groups, where a single question can be studied in depth [9]. The initial step in the NGT process is that members of the group independently generate ideas on a specific topic in response to a pre-selected question. The nominated items from the group are then shared, discussed and clarified, and grouped thematically (as applicable) by the participants, and ultimately ranked by participant votes. This is a consensus technique that provides a structured format, ensures participation of all members and achieves ranked results, and has the additional advantage of providing both qualitative and quantitative data [9, 10]. Patient research partners within the OMERACT SIG have contributed in the domain selection process, and larger groups of patients have endorsed the proposed TJR outcomes by survey [7, 11]. Patient research partners comprise an important critical part of all OMERACT working groups. Patient panels comprising of only patients have been utilized in other settings [12]. To our knowledge, in-depth qualitative studies in patients who have undergone TJR are limited.. We sought to explore patients’ priorities directly.

The purpose of this study is to hold group discussions with patients who have received THR and TKR, and use an open ended question to elicit the most important outcomes after TJR from the patients’ perspective, and then rank the outcomes. We hypothesize that TJR patients evaluated with the NGT will rank the relief of pain, improved function, satisfaction with their surgical outcome, and quality of life the outcomes most important to them and validate their inclusion in the core domain set.

## Methods

Patients over the age of 18 who had undergone TKR or THR between 2016-2018 were identified by their surgeons. All participants were selected from the surgical practices of high volume orthopedic surgeons who operate at a high volume orthopedic hospital, where approximately 10,000 TKR and THR are performed each year. The collaborating surgeons sent a letter to each potential participant informing them of the study and inviting them to participate in the nominal groups. Patients who did not respond to the letter directly were contacted by telephone or e-mail, invited to participate and scheduled for a nominal group session. Two of 8 groups were purposefully sampled from patients under age 45, to examine whether the perspective of younger patients differs from those across the age range. We preferentially recruited from a practice with a high proportion of African-American patients to increase inclusion of African-Americans in the groups. All patients provided written and informed consent, and the study was approved by the institution’s ethical review board (approval received November 30, 2018; IRB #2018-2087).

Patient demographics including age, sex, education, race, marital status, and employment status were collected immediately prior to the nominal group after informed consent was obtained. As TJR outcomes in a high volume hospital typically include better outcomes and higher satisfaction, we wanted to be transparent about the characteristics of the study cohort [13, 14]. Patients therefore completed a questionnaire containing either the Hip Disability and Osteoarthritis Outcome Score(HOOS) Joint replacement(JR), or the Knee Injury and Osteoarthritis Outcome Score(KOOS) JR . The HOOS JR and KOOS JR are validated short form questionnaires that assess post-operative pain and function after arthroplasty [15, 16]. In addition, patients completed a questionnaire describing their satisfaction with their surgical outcome in four areas: pain relief, functional improvement for housework/yardwork, improving ability to do recreational activities, and overall satisfaction. Each area was answered in a 5-point Likert scale ranging from very satisfied to very dissatisfied.

NGT is a highly structured group discussion format derived from traditional focus discussion groups. NGT leads to a group consensus and permits the group to define their priorities in response to a specific question that is analyzed in depth. Using NGT, in the first step the participants independently consider and record their responses to a specific question. Next, in a round-robin fashion, each participant presents one idea at a time, and the ideas are recorded verbatim. This phase continues until no new ideas are generated by the group. The groups then discuss and clarify the responses; responses are grouped together where thematically appropriate. In the final step, ideas are ranked and prioritized. The major benefit of using NGT is the patient group can reach consensus; in this study the question of interest was to have post TJR patients discuss and determine the most important outcomes of TJR. NGT provides qualitative data acquired in an open ended manner that can subsequently be quantified by ranking. Themes are not selected a priori, but are determined by the group discussion as responses are grouped into themes. After completion, the responses were distributed into final themes that emerged in the groups as agreed on by the investigators.

The patient discussions lasted approximately 1 hour and were held in non-clinical conference rooms in the hospital. All groups were led by an experienced leader in NGT(JS, SG) assisted by a research assistant (SM). A question selected by the senior authors(JS, SG) was presented to the group after informal testing with patients in their clinics . All participants were asked to consider the question “What result/results matter the most to a patient undergoing/having a knee or hip replacement?”. Each participant was given a blank sheet of paper with the question written on it and asked to list as many items that they think of to answer the question. The answers were clarified if necessary, and the responses were recorded on an easel by the moderator. The group then discussed the responses, collapsing some responses together as themes emerged after discussion. The responses were ranked on index cards by allocating a score to the three most important responses, with 3 being the highest score; each participant had a total of 6 votes to distribute. The rank order was then determined by the total scores. The most important items received the most votes by the greatest number of participants.

The group discussions were held in January and February 2019 and were recorded for accuracy. The recorded discussions were subsequently reviewed for accuracy and completeness of the nominated responses. The comprehensive list of the patients’ statements were further divided into the themes identified during the group sessions, with agreement of the investigators. The sessions were continued until theme saturation was confirmed, and no new themes emerged [17].

## Results

**Fig. 1:**
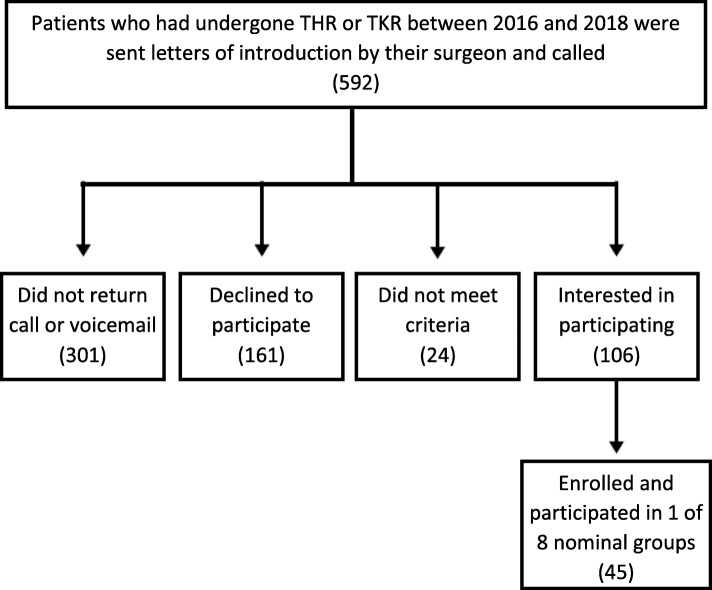
Recruitment Flow Chart

Eight nominal groups were held in January and February 2019. Of 592 patients who had undergone THR or TKR between 2016 and 2018 and were sent letters of introduction by their surgeon or called, 45 were enrolled and participated in 1 of 8 nominal groups (Fig. 1). After completion of the initial 6 groups and achievement of data saturation, the lack of young participants was noted and Groups 7 and 8 were convened with an additional 9 patients, purposefully drawn from patients <45 years of age. The mean(SD) age of NGT 1-6 was 71.1±9.3 years vs. NGT 7-8, which was 36.8±7.4 years **(**Table 1**)**. Groups included 15.6% African Americans, by purposely sampling a practice with a high proportion of African Americans, who comprise ≤4% of most arthroplasty cohorts [18, 19]. The scores on the HOOS JR/KOOS JR, demonstrated excellent pain and function (Table 2). For the patients who had undergone THR, >90% reported no/mild pain, and >90% reported no/mild difficulty in activities of daily living-function. For the patients who had undergone TKR, >79% reported no/mild pain and 1 patient reported severe pain. Function in activities of daily living was good, with >70% had no/mild difficulty. Satisfaction with pain relief and satisfaction with the improvement in the quality of life was also high among the group participants**(**Table 3**).**

**Table 1:** Baseline Characteristics

Baseline Characteristics	Total *N* = 45	Nominal Groups #1–6			Nominal Groups #7 & 8	
		TKR *N* = 19	THR *N* = 14	Both TKR & THR *N* = 3	TKR *N* = 2	THR *N* = 7
Age (mean, years, Standard Deviation)	64.4 ± 16.2	75.7 ± 8.2	65.9 ± 8.1	66.3 ± 5.4	32.5 ± 7.5	38.2 ± 6.7
Men, n (%)	20 (40.4)	6 (31.6)	8 (57.1)	1 (33.3)	1 (50)	4 (57.1)
Black, n (%)	7 (15.6)	3 (15.8)	3 (21.4)	–	–	1 (14.3)
Asian, n (%)	1 (2.2)	–	–	1 (33.3)	–	–
Hispanic, n (%)	2 (4.4)	–	–	–	–	2 (28.6)
Multi-race, n (%)	3 (6.7)	1 (5.3)	1 (7.1)		–	1 (14.3)
Education						
High school, n (%)	1 (2.2)	–	1(7.1)	–	–	
Some college, n (%)	5 (11.1)	1(5.3)	3(21.4)		–	1 (14.3)
Trade/technical/vocational training	1 (2.2)	–	–	–	–	1 (14.3)
College or above, n (%)	36 (80.0)	18 (94.7)	10 (71.4)	3 (100)	2(100)	3 (42.9)
Employment						
Employed for wages, n (%)	21 (46.7)	3 (15.8)	9 (64.3)	3(100)	2 (100)	4 (57.1)
Self-employed, n (%)	9 (20.0)	4 (21.1)	3(21.4)	–	–	2 (28.6)
A homemaker^a^	1 (2.2)	–	–	–	–	1 (14.3)
Out of work but not looking for work, n (%)	1 (2.2)	1(5.3)	–	–	–	–
Retired, n (%)	13 (28.9)	11 (57.9)	2 (14.3)	–	–	–
Reason for surgery						
Osteoarthritis, n (%)	34 (75.6)	13 (68.4)	12 (85.7)	3(100)	1 (50)	5 (71.4)
Rheumatoid arthritis, n (%)	3 (6.7)	2 (10.5)	1(7.1)	–	–	–
PsA, SpA, Lupus, n (%)	1 (2.2)	–	–	–	1 (50)	–
Other arthritis, n (%)	4 (8.9)	3 (15.8)	1(7.1)	–	–	–
Fracture, n (%)	1 (2.2)	–	–	–	–	1 (14.3)
Avascular necrosis of the bone, n (%)	2 (4.4)	1(5.3)	–	–	–	1 (14.3)

*THA* Total hip arthroplasty, *TKA* Total knee arthroplasty, *PsA* Psoriatic arthritis, *SpA* Spondyloarthritis

^a^someone who manages a home

**Table 2:** HOOS/KOOS Scores

HOOS/KOOS Scores	Overall score	Nominal Groups #1–8	
		None/Mild N (%)	Moderate/Severe/Extreme N (%)
HOOS (mean ± SD)	92.6 ± 12.3 (*n* = 23)	*n* = 24	*n* = 24
Pain: on stairs		23 (94.1)	1 (4.2)
Pain: walking on uneven surface		22 (91.7)	2 (8.3)
Function: Rising from sitting		22 (91.7)	1 (4.2)
Bending to the floor/pick up an object		23 (94.1)	–
Lying in bed		23 (94.1)	–
Sitting		22 (91.7)	1 (4.2)
KOOS (mean ± SD)	84.2 ± 16.8 (*n* = 19)	*n* = 24	*n* = 24
Stiffness		18 (75)	6 (25)
Pain: Twisting/pivoting		19 (79.2)	3 (12.5)
Pain: straightening knee fully		21 (87.5)	2 (8.3)
Pain: going up or down stairs		19 (79.2)	5 (20.8)
Function-Standing up		18 (75)	4 (16.7)
Rising from sitting		18 (75)	4 (16.7)
Bending to floor/picking up an object		17 (70.8)	4 (16.7)

Scores 1–100, higher is better

*HOOS* Hip disability and Osteoarthritis Outcome Score, *KOOS* Knee injury and Osteoarthritis Outcome Score

**Table 3:** Satisfaction Scores

Satisfaction Scores	Nominal Groups #1–8 *N* = 45	
Satisfaction with….THA- total hip arthroplasty, TKA = total knee arthroplasty, PsA = psoriatic arthritis, SpA = spondyloarthritis ^+^someone who manages a home	Satisfied (very/somewhat)	Neutral or Dissatisfied (very/somewhat)
Pain relief, n (%)	43 (95.6)	–
Ability to do housework or yard work, n (%)	41 (91.1)	1 (2.2)
Ability to do recreational activities, n (%)	40 (88.9)	3 (6.7)
Overall satisfaction, n (%)	40 (88.9)	–
Improve QOL, n (%)	38 (84.4)	3 (6.7)

***The question “What result/results matter the most to a patient undergoing/having a knee or hip replacement?”*** resulted in a ranked list of priorities **(**Table 4**).** A total of 216 votes was cast in NG 1-6, and 54 in NG 7-8, each participant had 6 votes to distribute among their top three choices Additional file 1 Table S1). The items were grouped into 5 themes. Overall, the most highly ranked items were 1) relief of pain, 117 votes, 2) improved function and mobility, 73 votes, 3) improved quality of life, 47 votes, 4) adverse events, 18 votes, 5) optimizing patient expectations/patient education 18 votes. Two subdomains, sleep comfortably and emotional impact aggregated within multiple themes.. Some patients relayed concerns about the process of TJR such as the ability to obtain rehabilitation and other post-operative care (5 votes). Other priorities were important to a smaller number of participants such as sleep, medication use or specific post-operative complications(worsening of diabetes, urinary retention, tachycardia/chest pain without cardiopulmonary complication), and were described separately.

**Table 4:** Question: “What result/results matter the most to a patient undergoing a hip or knee replacement?”

Domains	Total	NG 1–6	NG 7–8
NGT1–8, 45 people, 20 Male, 25 Female; 7 African-American, 28 White, 1 Asian, 2 Hispanic, 3 Multi Race; 270 votes	*n* (%)	*n* (%)	*n* (%)
A. Relief of Pain	117 (43.3)	98 (45.4)	19 (35.2)
B. Improved Function	73 (27.0)	64 (29.6)	9 (16.7)
C. Improved Quality of life including social and family participation	47 (17.4)	28 (13.0)	19 (35.2)
D. Avoiding Adverse Events/Revision Surgery:	20 (7.4)	18 (8.3)	2 (3.7)
F. Optimization of post-operative care/Patient education	13 (4.8)	8 (3.7)	5 (9.3)
Total votes:	270	216	54

### Relief of pain

Relief of pain was the most highly ranked priority for NG 1-6, and achieved 98 of 216(45%) total votes. Similar to the older patients, NG 7-8 also ranked pain the highest, with 19 of 54(35%) total votes. In NG#1, a patient reported that he “woke up one morning unable to get out of bed, I was in total pain, I asked God what did you do to me”. Patients in NG#2 described the desired outcome “to be pain free”, and “not to have to think about it”, to “stand without pain” and “to eliminate pain overall”. In NG#3, one patient described “it goes beyond physical pain, it isolates you, you’re not living the way you’re used to living”. **Taking less medication** was included as a subdomain in the relief of pain theme. This theme received 29/216(13%) votes in NGT 1-6 and 9/54(9%) votes in NGT 7-8. Patients expressed the concern that they would be able to take less pain medication after surgery, a concern that was linked to relief of pain, and aggregated with the domain relief of pain during the discussion. A patient in NG#1 told the group “I had a good friend, years before I was thinking about it(THR) and he spoke to me on the phone and he was taking pain meds like M&Ms. He died because he mixed the wrong meds. I remember taking pain meds and the pain was so severe that I convinced myself I had not taken pain meds a few hours ago and took more pain meds and that’s when I realized I needed the surgery”. Patients wanted to take “less medication if at all”. Patients were concerned about pain management after surgery that was “So important in this day and age of opioid abuse and terrible outcomes of that”. A patient commented “I was eating Advil like they’re candy”. Patients in NG 7-8 wanted to “stop using Advil” and “stop using alcohol to eliminate the pain”.

### Improved function

This theme was described by the groups, receiving 64/216(29%) total votes in NG 1-6, and included mobility, motion, and strength, as well as the functional ability to perform activities of daily living and sports. For NG 7&8, function ranked third, with 9 of 54(16%) total votes. In NG#1, a patient described “I was able to walk, to dance, now I can go back to do it. Going back to normal routine”. In NG#2, patients discussed knee flexibility and strength, such as the ability to “lift objects without fear of my knee buckling”, to be able to “climb stairs without difficulty”, and to regain an “excellent ability to walk and exercise”. They noted that “Living in Manhattan all you do is walk, when you slow down, people bump into you, once someone had a dog on the leash; if you are too slow, someone with a push cart bumps into you; having two knees with the problem, it was difficult”, and “Being able to do what we need to do when we need to do it- its life”. One young patient mentioned “no more overcompensation for abnormal walking and pain”, and being able to walk long distances.

### Quality of Life (including social/family participation)

This specific theme garnered 28 /216(13%) votes in the older patients and 19/54(35%) of the votes among the younger patients. For younger patients in NG#7 and 8, quality of life including social participation was the second most significant outcome. In NG#2, patients described “Before I was captive to the pain and immobility, now I have an ability to be participating in life” and to “be involved with my kid’s activities” and achieve “normalcy in life”, and in NG#3 the patients described the expectation that they would be able to "return to normal life in all its aspects post-surgery”. A young patient in NG8 described being “an outcast in a group of friends, and now feels like a normal person her age”. Another patient described wanting to go out and “wear a bikini, I still want to feel normal”, and another that he “can finally walk to meet friends”. Another felt she could “Stop worrying about her future job or life logistics due to pain, and could think about marriage and kids”.

### Adverse events

This priority received 16/216(7%) votes in groups 1-6, and was less highly ranked among the younger patients 2/54(4%). Patients described wanting “everything to go alright during surgery, after surgery, my greatest fear–what if it got worse, infection”. Another patient mentioned “Coming out alive- not worse off than when you went in”. Revision surgery as a specific theme received 2 votes from the older population, the patient desired “longevity” of the TJR with “no repeated replacement of the joint in my lifetime”.

### Optimizing patient expectation/patient education of surgery results

This theme received 5/54(9%) votes from the younger patients and 13/216(6%) of the votes in the older group. A younger patient described having a “hard time my entire life, so it was huge to get answers….about what life will look like moving forward”. Another young man noted that he “had friends who didn’t understand and I needed a community who understood, where I could connect”.

#### Sleep comfortably

This subdomain received no unique votes, but was mentioned in 7% of the comments within the pain, function, and quality of life domains in NGT 1-6 and 16% in NGT 7-8, .A patient told the group **“**it kept waking me up, the pain before the surgery. My niece said to me I was crying in my sleep”**.** Patients desired the “ability to sleep again without prescription drugs or assistance”.

#### Emotional impact

The emotional impact described by patients was profound, aggregating with other domains that provided the context for the emotional response, but received no unique votes. Older patients in NG 1-6 included the emotional impact of relief of pain, improved function and improved quality of life in 60% of their comments, while the younger groups described emotional responses in 7% of their comments. Another patient said “I have 4 little grand kids and we’re on the floor all together wrestling – that’s very emotional; The thing is that I used to do it with my son and he likes to see me do it with his kids” and “one of the things that always keeps me going “. A younger patient described the emotional change from “losing the sense of depression caused by constant pain”.

## Discussion

This qualitative study using nominal group technique explored the question “What result/results matter the most to a patient undergoing/having a knee or hip replacement?”. To our knowledge, this is the first study asking patients to provide the TJR outcomes of importance to them in an open-ended manner. Previously, patients have been queried whether they concur with expert-selected outcomes determined by literature review or survey. Although the themes that emerged were concordant with the themes previously identified by the OMERACT TJR working group, this was not known a priori and therefore this study fills an important gap in domain selection for a patient reported outcome measure.

Most participants had undergone TJR for osteoarthritis, and most were very satisfied with the results of their surgery. Women and African Americans were well represented in the groups. African Americans comprise ≤ 4% of most arthroplasty cohorts [18, 19], so we invited participants from a practice with a high proportion of African-American patients to ensure good representation of this group. The participants were highly educated; >90% had some college education or above.

The results of this study confirm that TJR patients of all ages think that alleviation of pain is the most important outcome after TJR. For the older groups, improved function was the next most important, while the younger participants ranked improved quality of life and social participation higher than improved function. Concerns about adverse events and complications were also important to these patients, but ranked significantly lower for all ages. These findings, using an open ended qualitative research technique, are concordant with the domains selected by the expert-led OMERACT TJR SIG, that determined that pain, functional improvement, satisfaction and improved quality of life, and adverse events were core domains that should be included in all TJR clinical trials [7].

The OMERACT core domain set was determined by extensive surveys and systematic literature review, and has been endorsed by both patients and surgeons, but there was no open-ended qualitative research with patients contributing to the initial domain selection process [7, 11, 20]. Surgeons are important stakeholders in TJR outcomes, and the level of pain and functional impairment reported by patients is an important factor in surgeon's recommendation for TJR [21]. The patients’ perspective has been under reported, and should be included if patient satisfaction after surgery is important. This study confirms the importance of pain relief, functional improvement, improved quality of life, and avoidance of adverse events for patients after TJR using qualitative open ended techniques, and lends further support to the inclusion of measures of these domains in all TJR clinical trials.

The mean age of the first six nominal group participants was over 70 years, so we added 2 groups of patients under the age of 45 to explore the perspectives of younger patients(mean age,36.8±7.4), which is important as the mean age for people undergoing TJR is decreasing over time [22]. While the three most important outcomes were the same, improved quality of life and social participation was more important to the younger patients and ranked above function. Younger patients were more concerned that their friends were unable to understand their experience with chronic pain and disability, and that it was important to be able to discuss their priorities within a community who “understood”.

This study has certain limitations. While the participants were typical of TJR patients in age and sex, the educational attainment was very high, and may make these results less generalizable. In addition, all patients received their surgery in a tertiary referral hospital for musculoskeletal diseases that performs a high volume of joint replacement surgeries, whereas most patients receive their joint replacements in lower volume hospitals [23]. Patients receiving their joint replacements in high volume hospitals are more likely to be satisfied with their outcomes, so we included the survey results for clarity [24, 25]. 89% of the participants in this cohort reported that they were very satisfied with their pain relief, which may be higher than other post-operative cohorts. This may represent a selection bias among those willing to participate in the nominal groups, or reflect the experience in a high volume center, skewing the participants’ perceptions. However, we obtained and included questionnaire information so that the characteristics of the study cohort would be transparent. The low participation rates are nonetheless a limitation, although other studies using similar methods also had very low participation rates, with the exception being when the treating clinician recruited the patients directly(). We hoped that the direct introduction by letter from the surgeon would improve our ability to recruit, but participation remained low.

Several areas may contribute to overall patient satisfaction and have not, to our knowledge been described. Patients described the emotional toll of both their pre-operative disability and sense of isolation, and the emotional impact of their post-operative improvement, mentioning effects on both self-confidence and self-esteem. As one patient put it “I got my mojo back”, referring to sexual intimacy. Another described the importance of “wearing pretty shoes again” contributing to her self-image.

In summary, this study assessed the outcomes most important to patients after hip or knee replacement surgery. We confirm that patients prioritized relief of pain, improved function, restored quality of life and the avoidance of adverse events when considering hip or knee replacement surgery.

## Conclusion

Relief of pain and restoration of function, and improved quality of life are the 3 outcomes ranked highest by patients, confirming their inclusion in TJR clinical trials. These are the domains that are most important to both patients and clinicians, and should be measured routinely in all TJR clinical trials.

## Supplementary information

**Additional file 1 Table S1.** Appendix – Question: “What result/results matter the most to a patient undergoing a hip or knee replacement?”.

## Data Availability

The datasets used and/or analyzed during the current study are available from the corresponding author on reasonable request.

## Abbreviations

HOOS JR: Hip Disability and Osteoarthritis Outcome Score Joint replacement
JS: Jasvinder Singh
KOOS JR: Knee Disability and Osteoarthritis Outcome Score Joint replacement
NG: Nominal Group
NGT: Nominal Group Technique
OMERACT: The Outcomes Measures in Rheumatology
PsA: Psoriatic Arthritis
QOL: Quality of Life
SD: Standard Deviation
SG: Susan Goodman
SIG: Special Interest Group
SM: Serene Mirza
SpA: Spondyloarthritis
THR: Total Hip Replacement
TJR: Total Joint Replacement
TKR: Total Knee Replacement
