# Supplementary material for: Patients’ perspectives of outcomes after total knee and total hip arthroplasty: a nominal group study
Source: BMC Rheumatol. 2020 Jan 13;4:3. doi: 10.1186/s41927-019-0101-8 (PMC6956500; doi:10.1186/s41927-019-0101-8)
Supplement: Supplementary file 1 — Additional file 1 Table S1. Appendix – Question: “What result/results matter the most to a patient undergoing a hip or knee replacement?”. [file 41927_2019_101_MOESM1_ESM.docx]

Appendix Table 1– Question: “What result/results matter the most to a patient undergoing a hip or knee replacement?”

| Theme | Score |
| --- | --- |
| **NGT1, 6 people, 3 Male, 3 Female; 1 African-American, 4 White, 1 Asian, 36 votes** |  |
| 1. **Pain:** No more pain    - Great days and really severe pains, walking without a limp, two days later walking like a 90 year old man with a limp.    - Pain because I work in hospital setting in public affairs and when they see you’re at a certain age and you’re up against the wall they ask you what’s going on. Both the physical and mental part.    - I agree with the mental part because I used to walk like I was 90    - I woke up one morning I could not get out of bed, I was in total pain I could not get out of bed. I asked god what did you do to me. I could not get our bed, I struggled, it was impossible to go down the stairs. I took    - I had a good friend, years before I was thinking about it and he spoke to me on the phone and he was taking pain meds like M&Ms. He died because he mixed the wrong meds. I remember taking pain meds and the pain was so severe that I convinced myself I had not taken pain meds and took pain meds and that’s when I realized I needed the surgery.  - *Alleviating the pain* - *No pain* - *I have no pain* | 13 |
| 1. **Function:** Going back to a normal routine    - Going back to normal life before the onset of pain. The pain is an obstacle.  - *To be mobile to do anything in normal pursuit of life (3)* - *Walking, being not worrying about a flight of stairs, a big hill. Being mobile without pain.* - *Quality of life* - *Was able to walk, to dance, now I can go back to do it. Going back to normal routine.* - *Making sure life can continue as it did before* - *Being able to walk* - *I have unlimited walking ability* - *Well-being, feeling better about everything* | 12 |
| 1. **Quality of life:** Make sure I didn’t have to ask for help  - Wanted to be more independent, affect quality of life, quality of life is being independent - Feeling the pain was feeling handicapped - *Not feeling dependent on anyone* | 3 |
| 1. **Function:** Regaining range of motion  - *3 days a week at a desk/sitting for long periods of time getting up and down without pain* - *Riding my motorcycle and bicycles* - *Snowboarding again* | 3 |
| 1. **Pain:** Less (pain) medication if at all | 3 |
| 1. **Adverse Events:** Not feeling the fear of infection or set back  - Wanting everything to go alright during surgery, after surgery, greatest fear – what if it got worse, infection. | 2 |
| **NGT2, 8 people, 3 Male, 5 Female; 1 African-American, 6 White, 1 Asian, 48 votes** |  |
| 1. **Pain:** To be pain free  - Not to have to think about it - Made feel old - Made feel younger - *No pain in joint* - *Freedom from pain* - *Stand without pain* - *Less pain/no pain* - *I was in constant pain, is this going to go away, really high pain threshold and endured a lot of pain* - *Only I have pain when I bend the knee for a while* - *Ability to walk pain free* - *Have no pain* - *Eliminate pain overall* | 23 |
| 1. **Function:** Greater mobility    - Be able to move pain free    - Have joints react as they did before    - Moving becomes simple again  - *To be able to move more freely* - *Able to walk without pain* - *Climb stairs without difficulty* - *Excellent ability to walk and exercise* | 10 |
| 1. **Function:** Ability to participate in sports  - Cant to work out video, hip or knee would lock up - Can’t ride bicycle around central park - Replacement did not make me any faster - *Ability to exercise without pain* - *Being able to ride a bike* - *Being able to ice skate* - *Being able to ski* - *Resume athletics* - *Able to ride my bike on the street and park without pain* - *Engage in physical activity* - *Returning to the gym after a long absence* | 6 |
| 1. **Function:** Able to carry out ADL (activities of daily living)  - Before surgery had a hard time getting in and out of the shower, had a harder time getting around my parent and cooking - *Resume long walks* - *No stiffness when standing for more than one hour* - *Using public transportation, not falling down* - *Drive with greater confidence* - *Walking longer distances* | 4 |
| 1. **Quality of life:** Ability to have a normal life/quality of life  - Getting out of the shower, not falling down - *Ability to concentrate on things other than pain* - *Involved with my kids activities* - *Normalcy in life* - *Sleep better* - *About pain* - *Quality of life* - *Better life after surgery* - *Constant pain, too much pain before surgery* - *Before captive to the pain and immobility, now have an ability to be participating in life* - *Quality of life* - *Visiting family and friends outside New York* - *Travelling downtown to take classes without pain* - *Stay out of wheel chair for life* | 4 |
| 1. **Function:** Flexibility/Strength of joint  - Physical mobility - Range of motion - *Strength of the joint* - *Life objects without fear of knee buckling* - *Knee flexibility* - *Walking much better with other leg and climbing much better with other leg* | 1 |
| **NGT 3, 5 people, 2 Male, 3 Female; 1 African American, 4 White; 30 votes** |  |
| 1. **Pain:** Decreased or no pain  - Goes beyond physical pain, isolates you, not living the way you’re used to living - Emotional pain, to be free and feel like yourself again - When I was approaching the surgery, what expectations would I reasonable have after surgery? Degree of pain relief varies and this better set the expectation. - Concern about when I would be able to not require any pain med intervention - *What level of pain will I have after surgery* - *Degree or level of pain prior to surgery depending on how long condition lasted for you to want to undergo a surgical procedure.* - *Living with a lot of limiting pain, want to get to the end of the pain that you have to live with every day or get through rehab.* - *What is the statistical outcome of positive results?* - *Relief from pain* - *Over time, when I tried to ignore the pain, it got significantly erroneous that I would have to stop walking after a block.* - *Pain outcome* - *Walking without pain* | 13 |
| 1. **Quality of life:** What will my quality of life be post-surgery – Normal life  - Expected to return to **normal life** in all its aspects post-surgery - To have no physical limitations | 6 |
| 1. **Function:** Improved function  - Better walking, being able to walk wherever you have to go, being able to walk up and down the steps when I need to. Not taking cabs - Safety in my balance - Hoped to return to jogging and was disappointed when couldn’t - *Regained ability to walk, climb and squat* - *Improvement in mobility* - *Regain my overall fitness* - *How will my other joints be affected, knees/hips?* - *Return to activities, hiking exercise, dance* - *What impact will it have on my golf game* - *Able to exercise more intensely* - *Return to walking with an upright posture* - *How will my body react to the new hip* - *Ability to resume usual activities* - *Better motion of the hip joint and improved strength* - *Ability and confidence to perform my professional activities* | 4 |
| 1. **Pain:** Reduction in pain with activities of daily living and sleeping  - When you are worn out from coping with pain takes a lot out of you - Could not do public transportation, when I get home I am not physically exhausted but mentally exhausted. - Go to a normal work environment - Sleep issues need to be resolved | 3 |
| 1. **Adverse Events:** Long term good outcomes  - How long is the glue going to last - How long will the joint function - Will there be long term good function - IS there a warranty? - Durability of the implant - Being infection free - *How long is surgery effective* | 3 |
| 1. **Optimization of post-op care:** How long will the recovery be? – having a short recovery period  - Prepared for a lengthy recovery – was not an issue, expected a year - I expected the same, but was done in 6 weeks. - **Having a short recovery period** - *How long will I be out of work* | 1 |
| **NGT 4, 7 people, 3 Male, 4 Female; 2 African American, 5 White; 42 votes** |  |
| 1. **Pain:** Elimination of joint pain  - Was in constant pain, never was without pain - I assumed joint pain would go away after surgery, because it was the 2^nd^ best invention - Improved my mental health - You’re a slave to the joint - Gravity itself is your enemy - You can focus on other things instead of your knee or hip - *Pain elimination* - *Be free of pain when walking* - *Pain is physical, has to do with tissue, walking is exercise* - *You’re not going to be able to do that you want to do* - *Before my surgery I stopped and talked to someone, couldn’t move and needed surgery* | 19 |
| 1. **Function:** Freedom to do all activities  - Walking, biking, hiking travel, exercise. - It’s part of your life – like brushing your teeth can’t get on without it - Benefit, independence - Part of living – doing the activities you want to do - Strengthening, what you do is who you are - *Ability to walk without the cane* - *Full movement* - *Ability to try new things* - *Ability to try new things before surgery* - *Walk the dog without pain* - *Independence* - *Ability to be active again and ability to walk* - *Move without assistance* - *Travel to faraway places* - *Able to exercise more* - *Able to swim* - *Participation in gym activities* - *Minimum restriction* - *Walking long distance without stopping* - *Do exercises* - *Walk as long as I want to* - *Be free of pain when walking* - *Pain is physical, has to do with tissue, walking is exercise* - *You’re not going to be able to do that you want to do* - *Before my surgery I stopped and talked to someone, couldn’t move and needed surgery* - *Not curtail any activities* - *Shopping without assistance* - *Independent living* - *House chores* - *Travel independently* | 6 |
| 1. **Optimization of post-op care:** Post-operative care  - Not everyone has the where with all to find post-operative care of the best caliber due to many different reasons. (location, insurance) - Just as important as - *Whether or not to hire or get a private room and nurse and rehabilitation facility* - *Availability of MD after surgery* - *Access to rehab facility* - *The hospital have good networking with first two weeks of home healthcare company, accounts for itself, keep appointments* | 3 |
| 1. **Adverse events:** No infection  - It is always a reality - It’s a horrible thing, it’s a sin that happens - Staph - System, body anywhere – infection is an infection - It depends if you are at a rehab center right after, sometimes it’s from rehab - It’s a complicated thing, depends on the metal type | 3 |
| 1. **Function:** Flexibility or mobility  - If you’re completely locked up the reason you come here because you can come again - Something to worry about before and after the surgery – it can affect your sleep - The pain is so intense | 3 |
| 1. **Optimization of post-op care:** Physical therapy appointments made after arriving at home – rehab  - *The ability of the replacement to interact with surrounding muscle groups* | 2 |
| 1. **Revision:** Longevity, no repeated replacement of joint in my lifetime  - *The useful life of the replacement* | 2 |
| 1. **Patient Education:** Education on what to expect with healing  - Exercise beforehand, teaching yourself to sleep properly - Know what to expect after surgery - Must to post-op exercises | 2 |
| 1. **Quality of life:** Social activities  - When you’re in pain you don’t really have a social life, but after the surgery I was able to socialize with friends again - Emotional health - Balancing your life - Going to church, social and emotional - *Socialize without worry about pain or movement* - *Able to attend church more regularly* - *When I first had my surgery I was in a lot of pain and when I went I couldn’t stay there for more than 1 hour. After the surgery I was able to stay for the whole time* | 1 |
| 1. **Function;** Strength  - It’s helpful to strengthen before the surgery - Cardio exercise is very important | 1 |
| **NGT 5, 6 people, 1 Male, 5 Female; 3 African American, 3 White; 36 votes** |  |
| 1. **Pain:** No pain  - I had pain for 10 months before surgery, tried non-surgical approach, that’s when I realized I had to have the surgery. - Pain is not normal and its emotionally and physically draining - Pain is a big emotional impact - I wasn’t so pleasant to be around - To make up your mind to go to surgery - *Being pain free* - *Successful surgery* - Pain free - Going through everything, want it to be successful - Resuming activities - Coming out alive - Not worse off than when you went in - *Pain relief* - *No pain when I walk* - *Putting my socks on with no pain* | 18 |
| 1. **Function:** Resuming all activities  - Has some emotional impact – when I was able to put my socks on without pain for the first time I had tears in my eyes - Less dependent - *Being back to my normal lifestyle* - *Gaining mobility* - *Being able to take care of all my personal needs* - *Increased mobility* - *Being able to go up and down steps, not the way I had to do it, a few steps at the time* - *Getting in and out of cars, getting in a taxi, telling the driver it’s going to take me longer than usual* - *Doing everyday activities* - *Living in Manhattan all you do is walking, when you slow down having two knees it was difficult* - *Driving without problem* - *Being able to do what we need to do when we need to do it- its life.* - *Spontaneously getting up.* - *Going shopping* - *Climbing stairs* - *Travelling going on all the tours* - *Dance* - *No more limping* - *Successful surgery* - *Pain free* - *Going through everything, want it to be successful* - *Resuming activities* - *Coming out alive* - *Not worse off than when you went in* | 12 |
| 1. **Quality of life:** A positive effect on my overall health  - Don’t have the worry or the stress - If you’re not able to exercise, your labs and everything gets screwed – to be able to exercise - You can do without think about doing - Walking longer distance - Running for a bus - Emotional component – say to yourself I’m better and I can do this. Had a granddaughters bat mitzvah, I’m doing this I can do this. - *Improved emotional state* | 4 |
| 1. **Adverse Events:** Good results for a long time  - Once you feel okay you want to continue to feel that way – 10, 20, 30 years. - No pain, activities being back to normal, overall health being back to normal | 1 |
| 1. **Adverse Events:** Good follow up care | 1 |
| **NGT 6, 4 people, 2 Male, 2 Female; 4 White; 24 votes** |  |
| 1. **Quality of life:** Return to quality of life  - For me there were things I could not do and that affected my quality of life, walking. I always had confidence in my body because I was an athlete and this made me lose my confidence - Playing with my grandchildren - It really affected my self-esteem - It has a sneaky way of getting to your self-esteem when you’re not included in groups that require quick physical movements - For me it was all the above, your daily routine is so impacted from the pain, it was just getting through the day. - *Restoration mobility, elimination of pain* - *If you’re used to being active you find that you’re restricted in a lot of the things that you like doing, you can’t walk, can’t go long distances. After a while if you have severe pain you learn not to do them.* - *Can’t move around a lot – I have meetings, travel for work.* - *Walking without aids* - *Getting function back* - *Returning to work out/exercises* - *Returning to exercise even if maybe on a lesser level* - *Transportation, functionality ease – getting in and out of a car, disembarking a plane that has stairs that roll up; walking through large train station to catch trains* - *Feeling ownership of my body* - *Has an* athletic thing to it, you’re used to your body doing what you want it to do - *Confidence in your body* - *Which activities would be restricted?* - *Ownership of body, confidence, body, all the things you couldn’t do that you want to do* - *Able to sit in a movie theater for 2+ hours* - *Hoping that the replacement does not feel like one* - *Ability to lift kids, my dog and objects* - *Return to cycling* - *Return to normal intimacy/sex* - *Walking miles without having to stop to rest* - *Chasing my grandchildren around* - *Less radiological testing and shots* | 9 |
| 1. **Adverse Events:** Successful surgery meaning no problems during and after surgery  - No complications - No wound complications - No short term or long term - *Length of recovery/rehab* - *Rapid discharge from the hospital* - *Wanted to stay longer, wasn’t ready to leave* - *Doctor follow up* - *Having a smooth pre-surgical and post-surgical continuum* - *Confidence in the surgeon and the surgeon’s practice* | 6 |
| 1. **Pain:** Relief from pain  - I couldn’t function, everyday your sleeping, walking climbing stairs, it was trickled down to everything that basically it consumes your whole being - I was eating Advil like they’re candy - How do you deal with the pain is a big thing - You just wanted to be free of it at the same time very nervous about it. - *Movement without pain that is similar to that before pain started* - *Movement without pain* | 3 |
| 1. **Pain:** Pain after surgery  - Pain after second surgery was significantly less than the first one. - Different | 3 |
| 1. **Function:** Elimination of all the questions about how you feel – I didn’t want hip/knee problem to define me/disability  - People are constantly asking you questions – am I alright - Patience issues - It starts to define you - You don’t want to talk about it all the time - *Less doctor visits* | 2 |
| 1. **Quality of life:** Wearing pretty shoes again, maybe even a heel  - Feels good - Self-image - Have to think about what kind of shoes you can wear, proper footwear - Footwear issue | 1 |
| **NGT7; 3 people; 2 Male, 1 Female; 3 White; 18 votes** |  |
| 1. **Pain:** Eliminate pain  - Pain while walking, sitting, sleeping - *Stop using Advil and pain meds* - *Stop using alcohol to eliminate pain* | 9 |
| 1. **Function:** Mobility  - Going up and down stairs, squatting on toilet, walking - *Increase range of motion in my hip* - *Bend down to floor without pain* | 3 |
| 1. **Quality of life:** Continue activities  - Exercise - Do more activities - *To be able to lift weights and kids without pain* | 2 |
| 1. **Quality of Life:** Improve quality of life  - Improve overall daily function - Climb stairs - *Did not want to miss any more work – miss a lot with pain management and going to physical therapy* - *Walk all day* | 2 |
| 1. **Adverse Events:** No complications  - Heard of things happening, like infections | 2 |
| **NGT8, 6 people, 3 Male, 3 Female; 4 White; 36 votes** |  |
| 1. **Quality of life:** Return to pre-activity level prior to surgery  - Participate in sports and recreation and be able to exercise again - Wear heels again - Be able to perform activities for lifestyle typical of patients age - Walk long distances and be able to get around - Resume and feel safe when performing normal daily activities - Independence and be able to take care of one’s self - Be able to get around quickly in the event of emergency | 11 |
| 1. **Pain:** Eliminate Pain  - Have complete or partial pain relief - Pain while walking - Not be so limited overall | 10 |
| 1. **Optimization of post-op care:** What’s going to happen moving forward  - Have all questions and concerns clearly addressed before going into surgery - Understand plan for rehab after surgery - Have a community and support system throughout the entire process - Get an answer to the long term pain and discomfort patient experienced - Feeling content with and trusting the team of medical staff - Quality pain management post-op | 5 |
| 1. **Function:** Normal walking  - Be able to walk normally - Walk with comfort | 3 |
| 1. **Function:** Maintaining integrity of surrounding joints and muscles  - No more overcompensation for abnormal walking and pain - Stop worrying about general health and safety due to injury - Avoid future injuries due to compensation - Regain normal body alignment | 3 |
| 1. **Quality of life:** Discovering self without pain – psychological  - Have an understanding of true pain threshold - Personal change regarding self and tolerance - Understand and enjoy the benefits of life without pain - Stop worrying about future job or life logistics due to pain (marriage, kids) - Loose the sense of depression caused by the constant pain, emotional change | 2 |
| 1. **Quality of life:** Physical appearance  - Get back to physical appearance before pain impeded daily lifestyle - Having an ascetically pleasing, small scar or minimal scaring - Not having scar make patient feel self-conscious around peers | 2 |

| A-Pain |
| --- |
| B-Function (including flexibility and strength) |
| C-QOL/Social participation |
| D-Adverse events |
| E-Revision |
| F-Optimization of post-op care, recovery and rehabilitation |
| G-Patient education/optimizing patient expectation of surgery results |
